# eHealth Literacy: Extending the Digital Divide to the Realm of Health Information

**DOI:** 10.2196/jmir.1619

**Published:** 2012-01-27

**Authors:** Efrat Neter, Esther Brainin

**Affiliations:** ^1^Behavioral Sciences DepartmentRuppin Academic CenterEmeq HeferIsrael

**Keywords:** eHealth literacy, digital literacy, health literacy, digital divide, health information search

## Abstract

**Background:**

eHealth literacy is defined as the ability of people to use emerging information and communications technologies to improve or enable health and health care.

**Objective:**

The goal of this study was to explore whether literacy disparities are diminished or enhanced in the search for health information on the Internet. The study focused on (1) traditional digital divide variables, such as sociodemographic characteristics, digital access, and digital literacy, (2) information search processes, and (3) the outcomes of Internet use for health information purposes.

**Methods:**

We used a countrywide representative random-digital-dial telephone household survey of the Israeli adult population (18 years and older, N = 4286). We measured eHealth literacy; Internet access; digital literacy; sociodemographic factors; perceived health; presence of chronic diseases; as well as health information sources, content, search strategies, and evaluation criteria used by consumers.

**Results:**

Respondents who were highly eHealth literate tended to be younger and more educated than their less eHealth-literate counterparts. They were also more active consumers of all types of information on the Internet, used more search strategies, and scrutinized information more carefully than did the less eHealth-literate respondents. Finally, respondents who were highly eHealth literate gained more positive outcomes from the information search in terms of cognitive, instrumental (self-management of health care needs, health behaviors, and better use of health insurance), and interpersonal (interacting with their physician) gains.

**Conclusions:**

The present study documented differences between respondents high and low in eHealth literacy in terms of background attributes, information consumption, and outcomes of the information search. The association of eHealth literacy with background attributes indicates that the Internet reinforces existing social differences. The more comprehensive and sophisticated use of the Internet and the subsequent increased gains among the high eHealth literate create new inequalities in the domain of digital health information. There is a need to educate at-risk and needy groups (eg, chronically ill) and to design technology in a mode befitting more consumers.

## Introduction

eHealth, a relatively new concept [1], refers to “the use of emerging information and communications technology to improve or enable health and health care” [2]. eHealth literacy, which includes the component of *health literacy* [3-5], effectively links health consumers to the outcomes typical of Internet use—that is, opportunities, possible harm [6], and inequalities (eg, being part of a minority or disenfranchised group [7-9], education [10-13], age [13-15], and gender [15-17]). In the 1990s, the concern over inequalities related to the digital divide focused mainly on infrastructural access: ownership, availability, and affordability of the infrastructure [18]. The discourse on the digital divide has expanded to other concerns, shifting the emphases to patterns of access [19], usage [20,21], and online skills rather than mere access to technology [13,20,21]. eHealth literacy may constitute a second divide in the health domain [21,22].

Norman and Skinner [23] propose that eHealth literacy is “the ability to seek, find, understand and appraise health information from electronic sources and apply knowledge gained to addressing or solving a health problem.” They propose that eHealth literacy encompasses 6 kinds of literacies: *traditional* (literacy and numeracy), *information*, *media*, *health*, *computer*, and *scientific*. Of these, media and computer literacies are unique attributes of the Internet context, with media being the awareness of media bias or perspective, the ability to discern both explicit and implicit meaning from media messages, and deriving meaning from media messages. The literature includes other ways in which perceived capability or efficacy was measured, but these were not specific to health information on the Internet [24-26].

Norman and Skinner [23,27] advocate matching eHealth technologies to the skills of their intended users. Such a fit can be realized by improving users’ working knowledge of computers (or of the particular language or skill) to a level conducive to achieving health-related goals, as well as by designing systems with the users in mind. To further address this divide, Norman and Skinner developed an eHealth literacy scale (eHEALS) to measure eHealth literacy [27], using a sample of Canadian adolescents. They emphasized that eHealth literacy should be viewed as a process that evolves over time, while the pace of development depends on technology and context (personal, social, and environmental), rather than on a static attribute. Viewed as malleable, eHealth literacy may indeed “empower individuals and enable them to fully participate in health decisions informed by eHealth resources” [23]. Conversely, the extension of digital resources to the health domain in the form of eHealth literacy can also create new gaps between health consumers [14,28]. The digital divide between the haves and have-nots [29] appears to be closing in developed economies in terms of access to the medium; nevertheless, eHealth literacy hinges not on the digital divide but rather on the knowledge gap [30], thus lending support to the hypothesis that information technology is creating a new social inequality, rather than leveling out social discrepancies [28,31].

New inequalities may surface with use of the Internet. Although most people still prefer to receive health information verbally through face-to-face contact with practitioners [12], those with better digital and health knowledge can be expected to consume more information [28] in various forms (whether written or not), and even more so when information is written. Extensive use of digital resources may be associated also with the ability to employ a greater number of search strategies and with a clearer cognizance of the quality of, potential gaps in, and inaccuracies in the obtained information [32]. Finally, the outcomes and benefits of using the Internet for health purposes may extend the traditional outcomes of health literacy [33-36] by providing new areas of physician–patient interaction [37] and self-care.

### Present Study and Hypotheses

The present study focused on eHealth literacy and related it to the process and outcome of the information search. First, we examined the structure of the eHealth literacy concept to determine whether the 1-factor structure of the concept, as described by Norman and Skinner, is replicated also in the current study, which used a sample from another culture (Israel) and expanded the age range (compared with the adolescent sample of the original Canadian study [27]). Next, we examined the associations between eHealth literacy and issues related to the digital divide: the factors considered include sociodemographic characteristics, Internet access and digital literacy, the processes involved in information consumption, and the outcomes of using the Internet for health information.

As the sample used by Norman and Skinner was age restricted, we posited no hypothesis about whether our age-expanded sample would replicate the structure of the eHealth scale in the Canadian sample. Following the literature on the digital divide and the digital divide index (DIDIX) in an Israeli sample [15], we hypothesized that people with higher eHealth literacy would be younger and of higher socioeconomic status, would have more access to digital resources, and would exhibit a higher degree of digital literacy than those with lower eHealth literacy.

Following the digital divide literature, we hypothesized the following in regard to the domain of information consumption. People with high eHealth literacy, compared with people low in eHealth literacy, would (1) use more sources of information (magazines, books, television and radio, and interpersonal resources), (2) use a variety of search strategies in addition to googling, (3) judge the information on the Internet more critically and would use more criteria for evaluating health information, and (4) experience more outcomes and in a higher valence as a consequence of using the Internet.

We did not hypothesize about a relationship of eHealth literacy with gender due to shifting findings. Losh [16] and Ono and Zavodny [38] found that gender inequalities in Internet access and usage either diminished, disappeared, or became very specific and context dependent; for example, findings indicate that gender differences shifted to other dimensions such as autonomy of use, experience, skill, and types of uses [17,39,40]. Additionally, findings in an Israeli sample indicate that the DIDIX was lower along gender lines than in any of the other characteristics studied (such as education, income, and age) [15]. We purposely did not present a hypothesis regarding eHealth literacy and health status due to conflicting findings in the literature (eg, Fox [41] vs Bundorf et al [42] on chronic illness; and Goldner [43] vs Wangberg et al [44] on perceived health).

## Methods

### Data Collection and Sample Characteristics

The current study was conducted as part of a larger study examining gaps between users’ needs, proficiencies, and usage processes, on the one hand, and eHealth resources in terms of quality, language, and assumptions regarding users, on the other hand. Data analyzed in this study were collected from a nationally representative random-digital-dial telephone household survey of the Israeli adult population (18 years of age and older) conducted from May to August 2008 (landlines only). At the time the study was conducted only 7.1% of the Israeli population owned only a mobile phone. Thus, we expected the landline sampling frame to adequately represent the Israeli adult population.

The sampling procedure through which the random digital dialing worked began by dividing statistical areas into 4 layers according to (1) population groups (Jews, Arabs, and mixed localities), (2) 7 geographical districts, (3) different sizes of settlements (big cities to small towns and villages), and (4) socioeconomic status index based on the Israeli Central Bureau of Statistics classification.

Sampling employed a dual-frame design, incorporating two selection stages without stratification in either frame. The larger frame was designed to provide national coverage of the eligible population. Calls were placed to 4286 residential households to identify 2201 eligible potential respondents who use the Internet. Of these respondents, 1289 used the Internet for health purposes. The interviews were conducted by professional interviewers who went through a special training session to familiarize them with the questionnaire’s terminology. The interviewers conducted the telephone survey using computer-assisted telephone interviewing software.

A comparison with the Israeli census data indicates that the survey sample in the current study is representative of the Israeli population in terms of sex and age distributions [45]). The survey sample was further controlled to correspond to regional population distribution (see Multimedia Appendix 1).

### Measurements

*eHealth literacy* (perceived) was examined using Norman and Skinner’s eHEALS scale [27]. The original scale was composed of 8 questions. Since 2 of the items of the original scale were tapped in our survey in a more detailed fashion, we used only 6 questions. The final scale in our study included 6 items, which met a satisfactory internal consistency criterion (alpha = .86). A 5-point response scale was used (from strongly agree to disagree). Respondents were assigned into two groups on the basis of the mean score they obtained for this 6-item scale of perceived eHealth literacy. The mean score on the scale was 3.34 (SD 0.88). We used the median score of the scale (median 3.4) to create two groups: those with a high mean eHealth literacy score (median ≥3.4); and those with a low mean eHealth literacy score (median ≤3.39).

*Internet access* was measured by asking participants whether they used the Internet in any of 5 locations at least once a month. A list with 5 locations was presented and participants indicated whether the option applied to them (at a library/community center, friend’s, neighbor’s, Internet cafe, or school/university).

*Digital literacy* was tapped by asking for the frequency of engaging in 6 activities (visiting blogs, participating in discussion forums, playing games, downloading or listening to music, downloading software, or emailing with friends). A 5-point frequency response scale was used (from very often to never). Additionally, the user’s perceived general Internet skills were tapped (not at all skilled, not very skilled, fairly skilled, very skilled, or expert) [22,46]. The total mean score for digital literacy was computed for each participant (alpha = .75).

Health information *sources* were examined by asking “How often do you get health information from the following sources?” A list of 6 health sources was presented and participants responded to each source. Apart from the Internet as a source of health information, the list included radio or television; newspapers/magazines or books; information obtained from a pharmacist, nurse, or physician; and information obtained from family members or friends. A 5-point response scale was used (from very often to never). A total mean score for the health information channels was computed for each participant (alpha = .64).

Health information *content* on the Internet was examined by asking “How often do you search the Internet for information related to the following domains and actions?” A list of 8 domains and actions related to health information was presented, and participants responded to each domain. The list included seeking information about physicians; institutions that provide health services (hospitals, community clinics, pharmacies, etc); potential treatments (procedures and drugs); and social support. A 5-point response scale was used (from several times a week to never). The total mean score for the seeking health information on the Internet was computed for each participant (alpha = .80).

*Search strategies* employed to obtain digital health information were examined by asking “In order to find health information on the Internet you usually do the following.” A list of 5 common search actions was presented: use a site that my physician recommended; follow links that appear on websites; ask questions in forums; use my Favorites list; and use a site that a friend recommended. A 5-point frequency response scale was used (from always to never). The total mean score for the health information search strategy was computed for each participant (alpha = .64).

*Evaluation criteri*a were examined using Barnes and colleagues’ [32] scale. Participants were asked how important the 5 following criteria were in judging a website: the purpose of the site is clearly stated and the information is accurate; it has a reliable source; a contact is available for questions/comments/help; retrieval is easy and can be done in a timely manner; and the scope of information suits my needs. A 5-point response scale was used (from very important to don’t know). The total mean score for the health website evaluation criteria was computed for each participant (alpha = .77).

*Perceived outcomes* of seeking health information on the Internet were examined by asking “Do you agree or disagree that seeking health information on the Internet...?” A list of 9 outcomes, adapted from Baker et al [47], was presented: improved your ability to manage your health needs; enabled you to ask your physician questions resulting from the information you acquired on the Internet; enabled you to show your physician the information that you retrieved; raised your sense of power in your encounter with the physician; improved your understanding of the symptoms, conditions, or treatments in which you were interested; updated your knowledge in health innovations; led you to take independent steps (such as seeing a specialist, or changing an exercise regimen or eating habits); enabled you to think about alternative treatment options; and made you more aware of patients’ insurance rights (all Israeli citizens have a health insurance). A 5-point response scale was used (from strongly agree to disagree). The total mean score for each participant’s outcome perception was computed (alpha = .87).

*Sociodemographic* information included sex, age, levels of obtained education, religiosity, perceived health condition, and chronic diseases.

*Perceived health* tapped respondents’ self-rated health, as compared with other people their age and gender. Respondents indicated whether they were about the average, somewhat above the average, much above the average, somewhat below the average, or much below the average.

The existence of *chronic diseases* was assessed by asking respondents whether they had any chronic diseases.

### Data Analysis

We first conducted principal components analyses on the eHEALS. Second, to indicate that our measures were separate factors, we conducted two sets of confirmatory factor analysis on all major variables, using the CALIS procedure of SAS version 9.2 (SAS Institute, Cary, NC, USA). Lastly, we used analysis of variance and χ^2^ to compare differences between groups, and Pearson correlation to examine relations between items or between indices.

## Results

### Exploratory Factor Analysis of the eHealth Literacy Scale

As the current study used only 6 of the 8 original eHEALS items, we conducted an exploratory factor analysis on these items. Principal components analysis produced a single-factor solution (eigenvalue = 3.551, 59% of the variance explained). Factor loadings ranged from .62 to .84 among the 6 items. Internal consistency reliability was analyzed on the 6 items, producing a coefficient alpha of .86, where item–scale correlations ranged from *r* = .50 to .73. These results are quite similar to those of Norman and Skinner [27], where the single-factor solution explained 56% of the variance, the internal consistency reliability was alpha = .88, and the item–scale correlations ranged from *r* = .51 to .76.

### Confirmatory Factor Analysis of the Research Scales

We calculated 2-model fit analyses to insure the assumption that each scale is independent of the other scales. In the first set of analyses, we used confirmatory factor analysis to test the structure of 4 scales: eHealth literacy, outcomes perception, digital literacy, and Internet access*.* The results confirmed that the 4 scales are independent of each other, and the 6-item scale used from Norman and Skinner’s [27] eHEALS is considered an independent scale.

In the second set of confirmatory factor analyses we tested the independence of an additional 5 scales in our study: health information sources, health information content, motivations for information search, search strategy, and evaluation criteria. The results confirmed that the 5 scales are considered independent of each other (see Multimedia Appendix 2).

### Sociodemographic Characteristics of the eHealth Literacy Groups

We examined the characteristics of the high and low eHealth literacy groups focusing first on the demographic variables of gender, age, and socioeconomic status. The high and low eHealth literacy groups did not differ in gender. There were 321 (50.7%) and 325 women (49.8%) in the high (n = 633) and low groups (n = 653), respectively (χ^2^_1_ = 0.11, *P* > .05). Likewise, the eHealth literacy score of men (mean 3.35, SD 0.89) and women (mean 3.31, SD 0.88) did not differ significantly (*F*_1,1284_ = 0.94, *P* = .332). However, the high eHealth literacy group was significantly younger (*F*_1,1284_ = 35.56, *P* < .000; mean 38.87, SD 14.40, years) than the low eHealth literacy group (mean 44.12, SD 17.00, years). The socioeconomic status of the high eHealth literacy group was also significantly higher than that of the low eHealth literacy group, as measured by education (mean score, on a 7-point scale, 3.99, SD 1.32 and mean 3.82, SD 1.33, for the high and low eHealth literacy groups, respectively, *F*_1,1274_ = 5.43, *P* < .02). There were 264 (41.9%) and 228 (35.2%) respondents with academic degrees, respectively, in the high (n = 630) and low (n = 647) eHealth literacy groups.

The health status of the eHealth groups was significantly different between the eHealth literacy groups. Respondents who reported that they were chronically ill had a significantly lower eHealth literacy score (*F*_1,1270_ = 8.87, *P* < .003; mean 3.19, SD 0.95) than respondents with no reported chronic illnesses (mean 3.37, SD 0.85). In addition, 164 respondents (25.3%) in the lower eHealth literacy group (n = 648) reported having a chronic illness, as compared with only 117 (18.8%) respondents in the higher eHealth literacy group (n = 624). The health status difference on eHealth literacy was independent of age: an analysis of variance on eHealth literacy revealed an insignificant interaction effect of health status and age (*F*_3,1262_ = 0.695, *P* =.44).

Health status was also examined in terms of perceived health. There was no significant difference between the high and low eHealth literacy groups in perceived health (*F*_1,1276_ = 0.432, *P* =.511). The high and low eHealth literacy groups reported similar self-rated health (mean 3.25, SD 0. 74 and mean 3.22, SD 0.68, for the high and low eHealth literacy groups, respectively).

### Internet Access and Digital Literacy

eHealth literacy emerged as related to digital access and literacy. Respondents in the high eHealth literacy group had significantly more access to computers and used the Internet more frequently than did the low eHealth literacy group (*F*_1,1281_ = 26.47, *P* < .001): the mean Internet accessibility score of the high eHealth literacy group was 6.19, as compared with a score of 5.86 among the low eHealth literacy group. Furthermore, the digital literacy reported by the high eHealth literacy group was significantly higher than that reported by the low eHealth literacy group: 2.67 and 2.24, for the high and low eHealth literacy groups, respectively (*F*_1,1280_ = 88.34, *P* < .001).

### Information Consumption: Health Information Sources, Health Information Content on the Internet, Health Website Evaluation Criteria

eHealth literacy is a marker for consuming more information, as displayed in Table 1. Overall, respondents in the high eHealth literacy group used significantly more information sources (*F*_1,1280_ = 11.01, *P* < .001) than did the low eHealth literacy group. Looking at individual items in Table 1, there is a significant difference between the two groups in their use of written material such as books, newspapers, magazines, and the Internet; there is no statistically significant difference between the two eHealth literacy groups in their use of live information from radio and television, a pharmacist, a nurse, or a physician (all *P* > .05).

**Table 1:** Scores for low and high eHealth literacy groups’ consumption of information on the Internet

Variable		Low		High		*F*	*P* value
		Mean	SD	Mean	SD		
Information source (index)		2.62	0.65	2.75	0.73	11.01	<.001
	Books	2.23	1.16	2.59	1.34	26.30	<.001
	Newspapers and magazines	2.64	1.14	2.87	1.22	12.40	<.001
	Internet	3.26	0.92	3.81	0.93	112.78	<.001
	Radio and television	2.81	1.13	2.79	1.23	0.68	.794
	Pharmacist	2.21	1.25	2.15	1.26	0.86	.355
	Nurse or physician	3.41	1.25	3.54	1.26	3.55	.06
Search quantity/variety (index)		1.75	0.59	2.05	0.73	66.28	<.001
Variety of search strategies (index)		2.16	0.74	2.59	0.90	87.08	<.001
Information evaluation (index)		4.29	0.68	4.53	0.50	52.21	<.001

Respondents in the high eHealth literacy group searched for significantly more content on the Internet (*F*_1,1280_ = 66.28, *P* < .001) than did the low eHealth literacy group, irrespective of the type of health content: social (eg, social support groups), service-related (eg, availability of services, or information on physicians, hospitals, and pharmacies), and therapy-related content (eg, health status, procedures, and medication).

The use of the Internet was different in terms of the search strategies employed by each of the two eHealth literacy groups. As can be seen in Table 1, those high in eHealth literacy used every strategy significantly more often than those low in eHealth literacy (*F*_1,1280_ = 87.08, *P* < .000). For example, they followed links, asked questions on Internet forums, followed recommendations of their friends and physicians, and used their Favorites list significantly more often than those low in eHealth literacy.

In addition, the use of the Internet by participants who scored high on the eHealth literacy scale was marked by significantly more scrutiny, caution, and evaluation of the information they retrieve (*F*_1,1280_ = 52.21, *P* < .000). Thus, for example, they looked for a contact address, wondered about the reliability of the source and the accuracy of information, and formed an opinion about the accessibility and availability of the information on the particular site they encountered. Information evaluation is related to eHealth literacy (*r* = .26, *P* < .000), but as the size of the correlation indicates, it is not synonymous with eHealth literacy.

### Outcomes of Information Search

Finally, those highly eHealth literate gained significantly more from their information search than did the low eHealth literacy group (3.40 and 2.76, for high and low eHealth literacy groups, respectively; *F*_1,1280_ = 177.76, *P* < .001). The results are displayed in Table 2. Cognitively, people in the high eHealth literacy group reported gaining a better understanding of their health status, symptoms, and optional treatments (see items in Table 2). They also benefited more instrumentally: the information search improved their ability to self-manage their health care needs, affected their health behaviors, and allowed them a better use of their health insurance. The benefits extended also to their interaction with the treating physician: they asked the physician significantly more questions than they would have without the digital information search, presented the physician with the information they retrieved, and felt significantly better positioned vis-à-vis the physician than did the low eHealth literacy group.

**Table 2:** Scores for low and high eHealth literacy groups in outcomes of information search

Variable		Low		High		*F*	*P* value
		Mean	SD	Mean	SD		
Outcomes (index)		2.76	0.88	3.40	0.83	177.76	<.001
	Understanding of symptoms, conditions, treatment	3.30	1.20	3.95	0.96	115.56	<.001
	Update in health innovations	3.01	1.24	3.71	1.16	108.04	<.001
	Self-managing health	2.37	1.24	3.13	1.34	87.39	<.001
	Affected health behaviors	2.75	1.25	3.41	1.25	87.39	<.001
	Use of insurance	2.23	1.33	2.77	1.43	45.95	<.001
	Asking physician questions	3.17	1.28	3.73	1.18	63.51	<.001
	Consulting physician on information retrieved	2.90	1.32	3.54	1.24	81.85	<.001
	Power position with physician	2.55	1.32	3.22	1.31	83.06	<.001

## Discussion

### Principal Results

The present study has demonstrated the utility of the eHealth literacy concept. Though we used only 6 of the original 8 items of the eHEALS, the 1-factor structure of the construct emerged also in the current Israeli sample, indicating that the concept of eHealth literacy is applicable to another culture and age groups.

The main contributions of this study, however, lie in demonstrating the relation between eHealth literacy and (1) the background attributes of the respondents, (2) patterns of information consumption, and (3) outcomes of the information search. In almost all 3 criteria, findings showed that the degree of eHealth literacy skills extended the digital divide into the health domain. The implication of these findings is that low eHealth-literate people would be limited in their use of the resources available on the Internet.

We hypothesized that respondents higher in eHealth literacy would be younger and more educated. This hypothesis was supported. We put forward no hypotheses regarding gender and health status (measured by perceived health and chronic illness), and the findings indicate no gender or perceived health differences; however, we noted a significant difference in health status: the chronically ill were lower in eHealth literacy. Hypotheses regarding greater digital access and higher digital literacy among those with high eHealth literacy were supported, as well as all of the hypotheses regarding information consumption: using more information sources, conducting more frequent and more varied searches, employing more search strategies, and evaluating the output. Moreover, as hypothesized, respondents higher in eHealth literacy used the information gained more than did respondents low in eHealth literacy. Indeed, for those who can realize the potential and possibilities, the Internet is a means of sustaining health, whether by providing information, linking to peers and professionals, or supporting self-management of health and illness [48]. However, the use of the Internet in the health domain is related to social inequality [49]: health information was already identified as capital-enhancing activity (vs recreational activity) [17,49], and the present findings indicate who among the connected benefits the most and in what ways. We found that differences in traditional variables of the digital divide literature (age, education, health status, digital access, and literacy) were associated with eHealth literacy, and we recorded new hypothesized differences in information consumption and outcomes gained from the use of the Internet. As theoreticians surmised [50,51], crossing the initial connectivity divide left numerous differences between people in how they incorporated the Internet into their lives.

### Comparison With Prior Work

The findings regarding the relationship between background characteristics and eHealth literacy are similar to findings obtained in studies documenting digital access and digital literacy disparities [13,20,22,30]. As expected, the eHealth literacy groups differed significantly in terms of education and age, duplicating differences found between those who have access to computers and the Internet and know how to use them [10,12,14] and those who do not know how to use computers and the Internet. Gender did not differ between our eHealth literacy groups, a finding congruent with others’ [16,38]. It demonstrates the conclusion that gender differences are highly contextualized. It is also congruent with Mizrachi et al [15], who found that the DIDIX along gender lines in an Israeli sample was the lowest among other characteristics (such as education, income, and age). Finally, and as hypothesized, the eHealth literacy groups were significantly different in digital access and digital literacy. Digital access avails the information search and digital literacy is conceptually a facet of eHealth literacy [23].

Health status was measured in our study in two ways: perceived health (ie, self-rated health) and reported chronic illnesses. Perceived health did not vary with eHealth literacy but the presence of chronic illnesses did, such that chronically ill respondents had lower eHealth literacy scores. The inconsistency between the two measures is acceptable, as the two assess different concepts; for example, a person may have a diagnosis of hypertension, but she may also feel healthier than most of her age group. Still, our findings on the two measures replicate some previous work [41,43] and contrast with others [42,44], and no clear picture yet emerges. It could be that the relationship between health status and information search is dependent on the health system. Indeed, Bundorf et al [42] suggest that searching the Internet for health depends on costs and benefits, such as paying out-of-pocket and opportunity costs; these vary across health systems and may explain the seemingly contradictory findings. The present findings of lower eHealth literacy among the chronically ill call for an empowering intervention on the part of service providers (for example, health maintenance organizations). The chronically ill population constitutes a highly distinguished group for service providers, and its eHealth literacy should be targeted for improvement. Planned learning experiences to improve their literacy in searching for, locating, evaluating, and using eHealth information is called for. As Hargittai [17] points out, “achieving a knowledgeable Internet citizenry is unlikely to be resolved through a solely technical approach that focuses only on infrastructure without any consideration of the social processes and institutions in which people’s Internet uses are embedded.”

The findings on consuming information—ways of searching, frequency of searching for various contents, evaluating the information, benefiting from the information—all demonstrate inequality among those high and low in eHealth literacy. All of these variables focus on utilization rather than on mere accessibility [52]; these variables exemplify new differentiated usage patterns among the connected that have the potential to contribute to social inequality [17,50,51,53,54]. These findings are more in line with the strong hypothesis than with the weak hypothesis of the digital divide. The strong hypothesis posits that “the emergence of the information society will create new social cleavages and strengthen old ones” [28], whereas the weak hypothesis claims that the new technology will level out old differences, admittedly after witnessing a temporary gap during the dissemination of the new technology. Still, it is possible that the weak hypothesis is not altogether amiss. We may be in the midst of a change, as exemplified in gender, which was in the past related to digital access and literacy and turned out to be unrelated to eHealth literacy in this study.

It is important to note that the fit or full utilization of technology depends not only on users and their characteristics but also on the technology itself. Technology may have different manifestations and affordances [55], and it could be tailored to fit different users [56,57]. The literature on informed choice demonstrates [58] that there are many ways of presenting information and choices, and it exhibits genuine efforts and achievements in presenting complex medical information to laypeople. Similar efforts are called for in the content, design, and ease of use of health information on the Internet, so that people low in eHealth literacy may make fuller use of the digital promise. The realization of such a promise may also call for setting standards and accreditation, and alerting consumers to the latter.

The results of the confirmatory factor analysis indicate that the concept of eHealth literacy is independent of related variables. Even the variable of *health website evaluation criteria*, which seems to measure media literacy (in this case, Web literacy), was found to be only moderately related to eHealth literacy (*r* = .26, *P* < .000). This may indicate that, as Norman and Skinner [23] point out, media literacy is only one aspect of eHealth literacy.

### Limitations

Our findings are hampered by 5 major limitations. First, we used only 6 items from the original eHEALS [27]. Even though the 1-factor structure of the concept, the internal reliability, and the item–scale correlations were highly similar to the original scale, the scales are not psychometrically equivalent. Second, the cross-sectional design of the study precludes causal conclusions and allows us to draw conclusions only regarding correlated relationships. For example, we can assume only that education is associated with eHealth literacy and not that it affects eHealth literacy. Third, we did not measure actual eHealth literacy but rather perceived efficacy of searching and using health information on the Internet. This limitation calls for an amendment. Measures of actual eHealth literacy are required. Indeed, there are measures for actual digital literacy [39,59] and there are measures for health literacy [60]. The two may serve as an inspiration for a measure that taps actual eHealth literacy. The fourth limitation is related to the third: our findings are based on self-reports and not actual performance or record of Internet use. More studies that measure actual use and skill [48] are needed. Finally, the fifth limitation is related to the landline sampling frame. This sampling frame excluded 7.1% of the population who owned only mobile phones at the time that the survey was conducted. These people may be younger and may have been more digitally and eHealth literate.

To conclude, the present study documented differences between respondents high and low in eHealth literacy in terms of background attributes, information consumption, and outcomes of the information search. The findings are mostly interpreted in line with the digital divide literature, replicating previously demonstrated relationships to background variables (demographics, digital access, and literacy), and identifying and documenting new cleavages (information consumption and perceived outcomes). The need to both educate at-risk and needy groups [61] (eg, chronically ill) and design technology in a mode befitting more consumers emerges. Addressing those needs may not diminish the digital divide altogether, but it may ameliorate its consequences by bringing more people into the have group.

## Multimedia Appendix 1

Characteristic of the participant’s sex and age compared with the population.

## Multimedia Appendix 2

Scales independence calculation through confirmatory factor analysis (CFA).

## Abbreviations

DIDIX: digital divide index
eHEALS: eHealth literacy scale
