# Supplementary material for: eHealth Literacy: Extending the Digital Divide to the Realm of Health Information
Source: J Med Internet Res. 2012 Jan 27;14(1):e19. doi: 10.2196/jmir.1619 (PMC3374546; doi:10.2196/jmir.1619)
Supplement: Supplementary file 2 [file jmir_v14i1e19_app2.pdf]

Multimedia Appendix 2: Scales independence calculation through confirmatory factor analysis (CFA)

The first CFA that tested the independence of four scales (eHealth Literacy, outcomes perception, digital literacy, and Internet access) yielded an acceptable fit level [64] of $\chi2(337, N= 1255) =1133$, $P< .001$, GFI= .94, AGFI=.92, Bentler-Bonett NFI=0.9, Bentler-Bonett Non-normed Index=0.92, and RMSEA =0.04. All the standardized factor loadings in the model were above .33. Inter-factor correlations were 0.09-0.68 ($P < .05$ for each of them). To validate the four-factor structure, we also conducted a CFA in which all items were allowed to load on one factor. The CFA yielded an unacceptable fit level of $\chi2(343, N=1255)=4408$, $P< .001$, GFI= .7, AGFI= .65, Bentler-Bonett NFI=0.63, Bentler-Bonett Non-normed Index=0.61, and RMSEA =0.097. Moreover, the AIC of 459 and SBC of -1271 of the four-factor model increased to 3722 and 1961, respectively, demonstrating the superiority of the four-factor model over the single factor model.

The second CFA that tested the independence of five scales (health information sources, health information content, motivations for information search, search strategy, and evaluation criteria) yielded an acceptable fit level [68] of $\chi2(419, N=1280)=2070$, $p< .001$, GFI= .9, AGFI= .9 , and RMSEA=0.05. But Bentler-Bonett NFI=0.8 and Bentler-Bonett Non-normed Index=0.8, both below the 0.9 accepted level. All of the standardized factor loadings in the model were above .38, and values of inter-factor correlations were 0.017-0.77 ($P < .05$ for those which were greater than 0.1). Modification analysis showed that three of the items were loaded on two factors. Modifying the model to include these three extra loads improved the fitting considerably. Now, $\chi2(416, N=1280)=1723$, $p< .001$, GFI= .91, AGFI= .9 , Bentler-Bonett NFI=0.82, Bentler-Bonett Non-normed Index=0.84, and RMSEA=0.05. Hence, modification improved only slightly the Bentler-Bonett and Bentler-Bonett NFI fit indices. To validate the independence of the five-factor structure, we also conducted a CFA in which all items were allowed to load on one factor. The CFA yielded an unacceptable fit level of $\chi2(425, N=1280)=442$, $P< .001$, GFI= .77, AGFI= .74, Bentler-Bonett NFI=0.56, Bentler-Bonett Non-normed Index=0.54, and RMSEA=0.086. Moreover, the Akaike information criterion (AIC) of 1232 and SBC of -928 of the five-factor model rose to 3592 and 1401, respectively, demonstrating the superiority of the five-factor model over the single factor model.
